# The Paradoxical Effects of Different Hepatitis C Viral Loads on Host DNA Damage and Repair Abilities

**DOI:** 10.1371/journal.pone.0164281

**Published:** 2017-01-04

**Authors:** Shu-Chi Wang, Kuan-Ru Lai, Chia-Yang Li, Chi-Shiun Chiang, Guann-Yi Yu, Naoya Sakamoto, Wen-Yu Tu, Meng-Hsuan Hsieh, Jee-Fu Huang, Wan-Long Chuang, Chia-Yen Dai, Ming-Lung Yu

**Affiliations:** 1 Health Management Center, Kaohsiung Medical University Hospital, Kaohsiung Medical University, Kaohsiung, Taiwan; 2 Changhua Christian Hospital, Changhua, Taiwan; 3 Graduate Institute of Medicine, Kaohsiung Medical University, Kaohsiung, Taiwan; 4 Center for Infectious Disease and Cancer Research, Kaohsiung Medical University, Kaohsiung, Taiwan; 5 Department of Biomedical Engineering and Environmental Sciences, National Tsing-Hua University, Hsinchu, Taiwan; 6 National Institute of Infectious Diseases and Vaccinology, National Health Research Institutes, Miaoli, Taiwan; 7 Department of Gastroenterology and Hepatology, Tokyo Medical and Dental University, Tokyo, Japan; 8 Hepatobiliary Division, Department of Internal Medicine, Kaohsiung Medical University Hospital, Kaohsiung Medical University, Kaohsiung, Taiwan; 9 Faculty of Medicine College of Medicine, Kaohsiung Medical University, Kaohsiung, Taiwan; 10 Graduate Institute of Medicine, Kaohsiung Medical University, Kaohsiung, Taiwan; 11 Center for Lipid and Glycomedicine Research, Kaohsiung Medical University, Kaohsiung, Taiwan; 12 Institute of Biomedical Sciences, National Sun Yat-Sen University, Kaohsiung, Taiwan; Harvard Medical School, UNITED STATES

## Abstract

Hepatitis C virus (HCV)-induced hepatic stress is associated with increased oxidative DNA damage and has been implicated in hepatic inflammation. However, HCV infection and replication are uneven and vary among individual hepatocytes. To investigate the effect of the viral load on host DNA damage, we used an Enhanced Yellow Fluorescent Protein gene (EYFP)-tagged HCV virus to distinguish between HCV intracellular high viral load (HVL) cells and low viral load (LVL) cells. The cell sorting efficiency was confirmed by the high expression of the HCV polyprotein. We found DNA damage γ-H2AX foci in the HVL population. Comet assays demonstrated that HVL was related to the extent of the DNA strand breaks. Surprisingly, the DNA qPCR arrays and western blotting showed that the damage-related genes GPX2, MRE11, phospho-ATM, and OGG1 were significantly up-regulated in LVL cells but inversely down-regulated or consistently expressed in HVL cells. The colony survival assay to examine the repair abilities of these cells in response to irradiation showed that the LVL cells were more resistant to irradiation and had an increased ability to repair radiation-induced damage. This study found that intracellular viral loads drove cellular DNA damage levels but suppressed damage-related gene expression. However, the increase in damage-related gene expression in the LVL cells may be affected by ROS from the HVL cells. These findings provide new insights into the distinct DNA damage and repair responses resulting from different viral loads in HCV-infected cells.

## Introduction

Hepatitis C virus (HCV) replicates in the cytoplasm and results in a chronic infection that may ultimately cause chronic hepatitis, cirrhosis, and hepatocellular carcinoma (HCC) [1]. In the general population, HCV infection precedes the development of HCC by 20–30 years [2, 3]. Early research has shown that HCV spreads via cell-to-cell infection and that HCV antigens appear to form large clusters [4]. However, most hepatocytes in a HCV-positive individual are not infected [5].

The level of mitochondrial oxidative injury in liver tissue may serve as an indicator of the extent of HCV infection [6]. Currently, the associations between viral and oxidative DNA damage responses are particular and increasing scientific interest. Viral replication within a host cell requires a large amount of exogenous genetic material, including DNA fragments and atypical structures. Recent reports have shown that the HCV core proteins diminish DNA repair [7], whereas the HCV E2-CD81 interaction induces double-stranded DNA breaks [8] and the HCV NS5A protein induces chromosome instability [9]. It is generally accepted that HCV viral replication induces DNA damage stress and activates DNA damage signal pathways that ultimately lead to apoptosis as part of the host cell immune surveillance defense. Sustained oxidative damage may contribute to the development of virus-associated HCC [10]; however, determining whether HCV perturbs this process and the involved mechanisms requires further investigation.

HCV serum viral loads were associated with an increased risk of developing HCC in a community HCV cohort study [11]. However, the serum viral load does not reflect the level of infected hepatocytes in the liver. Therefore, whether HCV-infected cells incur different viral load-dependent effects on host DNA damage or repair abilities requires further clarification. In the current study, we used fluorescence-activated cell sorting (FACS) and a fluorescence-tagged HCV virus [12] to differentiate between HCV intracellular high viral load (HVL, the population with the highest 20% EYFP intensity) cells and HCV intracellular low viral load (LVL, the population with the lowest 20% EYFP intensity) cells in a population of human hepatoma Huh7.5.1 cells *in vitro*. Using this model, we investigate the differential effect of the intracellular viral load on host cell oxidative damage and cell repair-associated gene expression.

## Materials and Methods

### Cell culture and HCV JFH1-EYFP infection

Human hepatoma Huh-7.5.1 cells (Apath, LLC, St. Louis, MO, USA) were cultured in Dulbecco’s modified Eagle’s medium with 10% heat-inactivated fetal bovine serum, 5% antibiotic–antimycotic and 5% nonessential amino acids. Huh7.5.1 cells were transfected with JFH1-EYFP particles (HCV cc, developed by Dr. Machi Yamamoto). At two days post-transfection, the culture media were collected and added to Huh7.5.1 cells for 6 h at a multiplicity of infection (MOI, focus forming unit per cell) of 0.01. Then, the infection medium was replaced with fresh regular culture medium. The infection proceeded for 5 days before harvesting for the experimental analysis [12].

### Distinguishing HVL and LVL cells from JFH1-EYFP-infected cells using a flow sorting system

JFH1-EYFP infected cells were analyzed by FACS (Beckman Coulter, CA, USA). The JFH1-EYFP-infected cells were detected in the FL1-EYFP channel using uninfected Huh7.5.1 cells as the control. The LVL cells were gated as the cell population with the lowest 20% EYFP intensity and the HVL cells were gated as the cell population with the highest 20% EYFP intensity in a dot plot. After sorting, the fluorescence intensity and the HCV core, NS3, and NS5A polyprotein expression levels were measured by western blotting to confirm the viral loads.

### γ-H2AX focus formation assay

After sorting, the JFH1-EYFP-infected cells were fixed with 4% paraformaldehyde and then incubated in 0.5% Triton-X100 on ice. The cells were blocked with 5% goat serum and stained with an anti-human phospho-H2AX (S139) eFluor® 570 antibody (ebioscience, USA). After staining, the slides were mounted in mounting medium containing DAPI (Vector laboratories, CA, USA). The total foci for each cell sample were counted using ImageJ software.

### Comet assay

The comet assay was performed using a Comet Assay kit (Trevigen Inc.) following the manufacturer’s protocol. Briefly, cell suspensions were harvested by centrifugation, and the cells were resuspended at 1 x 10^5^ cells/ml in ice cold 1X PBS (Ca++ and Mg++ free). The sample were electrophoresed in a neutral TBE buffer (89 mM Tris, 89 mM boric acid, 2 mM EDTA) to detect DSBs. Electrophoresis was performed in an alkaline solution (200 mM NaOH and 1 mM EDTA, pH > 13) followed by an unwinding solution (200 mM NaOH and 1 mM EDTA, pH > 13) to detect SSBs and DSBs. Dried samples were stained using a SYBR solution following the manufacturer’s instructions. The comet tail of each cell was quantified using Image-Pro Plus 6.0 software.

### q-PCR array and q-PCR of DNA repair genes

cDNA was synthesized using 1 μg of RNA and a High-capacity cDNA Reverse Transcription kit (Applied Biosystems, USA). A q-PCR array was designed using a primer design website (https://www.genscript.com/ssl-bin/app/primer) for a total of 48 DNA repair-associated genes and appropriate housekeeping genes in a 96-well plate format. The following genes were analyzed: 1) base excision repair (BER) genes (*APEX2*, *CCNO*, *LIG3*, *MPG*, *MUTYH*, *NEIL2*, *NYHL1*, *OGG1*, *PARP1*, *PARP3*, *POLB*, *SMUG1*, *TDG*, and *UNG*); 2) nucleotide excision repair (NER) genes (*ATXN3*, *BRIP1*, *CCNH*, *DDB1*, *ERCC1*, *ERCC2*, *ERCC3*, *ERCC4*, *ERCC5*, *ERCC6*, *ERCC8*, *LIG1*, *MMS19*, *PNKP*, *POLL*, *RAD23A*, *RPA1*, *RPA3*, *SLK*, *XAB2*, *XPA*, and *XPC*); and 3) other repair response genes (*ATM*, *EXO1*, *GPX2*, *MGMT*, *MRE11*, *RAD18*, *RFC1*, *TOP3B*, and *XRCC6BP1*). All the primer sequence shown in **S1 Table**. Power SYBR Green (Thermo Fisher, USA) PCR reactions were performed in triplicate for each sample and analyzed using the ABI Prism 7900 fast detection system. The housekeeping genes were used to normalize the data prior to analysis of the amplified transcripts with the threshold cycle (Ct) method using ABI 7900 system software.

### Western blotting

After the HVL and LVL cell populations were sorted and collected, the cells were lysed in ice-cold Tris buffer (50 mM, pH 7.4) containing 1 mM DTT, 1 mM EDTA, 150 mM NaCl, 0.25% deoxycholic acid, 1% NP-40, phosphatase inhibitor, and protease inhibitor (Calbiochem. Millipore) for 30 min on ice and then centrifuged at 13,000 x *g* for 20 min. The protein concentration was determined using the Bio-Rad protein assay. A total of 10 μg of protein lysate was resolved by 10% SDS-PAGE and transferred onto a nitrocellulose membrane. The membrane was blocked in 1X TBST containing 5% non-fat dried milk and then incubated with primary antibodies at 4°C overnight. The primary antibodies were against HCV NS3 (MAB8691, Merck Millipore, KGaA, Germany), HCV NS5A (MAB8694, Merck Millipore), HCV core (clone C7-50, MA1-080, Thermo Fisher, USA), CDK4 (clone DCS-31, C8218, Sigma-Aldrich), OGG1 (NB100-106, Novus Biologicals, USA), XPC, ATM (PA1-16503, Thermo Fisher), p-ATM (pSer1981, clone 10H11.E12, MA1-46069, Thermo Fisher), GAPDH (clone GAPDH-71.1, G8795, Sigma-Aldrich), and actin (clone AC-40, A3853, Sigma-Aldrich). The membranes were stained with HRP-conjugated secondary antibodies, and the signals were developed with chemiluminescence reagents (Amersham Biosciences, CA, USA). The chemiluminescent signal was captured by an ImageQuant™ LAS 4000 mini system (GE Healthcare Life Sciences).

### Colonogenic assay

The cells were irradiated in log phase using a cobalt source with a dose rate of 50 cGy/minute in the Department of Medical Imaging and Radiological Sciences, Kaohsiung Medical University, Taiwan. A total of 1 x 10^4^ cells were cultured for 24 h in a 35-mm dish containing 5 ml of medium, which was changed to a serum-free medium prior to cell irradiation. The cell numbers varied following irradiation; the cells were seeded in triplicate into 100 mm culture dishes containing 10 ml of cell culture medium and incubated for 10–14 days. Colonies were counted after staining with 3% Giemsa solution (Merck, KGaA, Germany) to determine the survival fraction [13].

### Statistical analysis

Data are presented as the mean ± the standard deviation (SD) for N ≥ 3 from three independent experiments. The statistical significance of the differences between two experimental measurements was assessed by Student’s T-test.

## Results

### Delineating LVL and HVL cells by fluorescence-activated cell sorting

**Fig 1:**
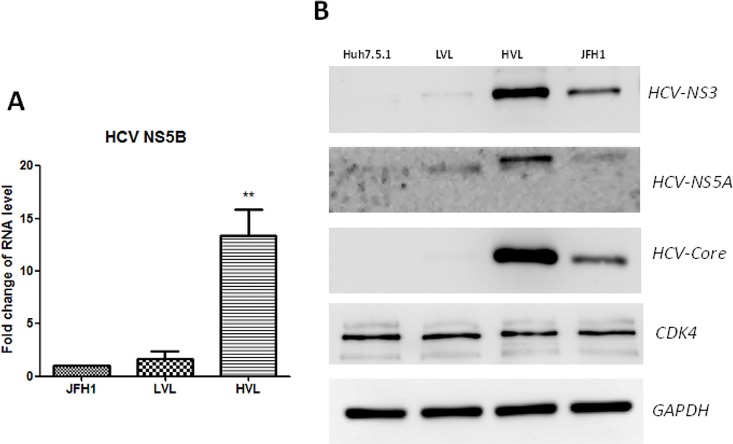
High expression of the HCV polyprotein in the HVL population. (A) HCV-NS5A q-PCR quantification of the sorted HVL and LVL cells. Data were normalized to the GAPDH housekeeping gene levels and presented as the fold change. **P < 0.01. (B) Analysis of several types of HCV viral polyprotein and the cell cycle CDK4 protein in uninfected Huh7.5.1, JFH1-EYFP, HVL and LVL cells on day 5 post-infection. GAPDH is the loading control.

JFH1-EYFP-infected Huh7.5.1 cells were collected on day 5 post-infection. The intracellular HCV viral florescence intensity of the infected cells was immediately analyzed by flow cytometry using two parameters in a dot plot displaying FL1-EYFP on the x axis and SSC on the y axis. For cell sorting, the cell population with the lowest 20% EYFP fluorescent intensity was gated as the LVL cells, and the cell population with the highest 20% EYFP fluorescent intensity was gated as the HVL cells (**S1 Fig**). The sorting efficiencies for the LVL and HVL cells were 94% and 89%, respectively. q-PCR confirmed that the HVL cells expressed significantly higher HCV-NS5B mRNA levels than the LVL cells and unsorted JFH1-EYFP infected control cells (*P* < 0.001) (**Fig 1A**). The western blot results demonstrated that the expression of the HCV core, HCV NS3, and HCV NS5A proteins was highest in the HVL cells, followed by the unsorted JFH1-EYFP infected cells, and was lowest in the LVL cells (**Fig 1B**). HCV proteins were not detected in the uninfected Huh7.5.1 cell lysates. PI staining confirmed cell viability after cell sorting (**S2 Fig**). There were no significant changes in cyclin-dependent kinase 4 (CDK4) expression for all cell populations, but the cell cycle assay showed that the HVL cells were slightly arrested in G1 phase compared to the total infected cells. (**S3 Fig**). These results confirmed that the HVL and LVL cells were successfully and efficiently sorted in this system.

### Host DNA damage and HCV viral loads

**Fig 2:**
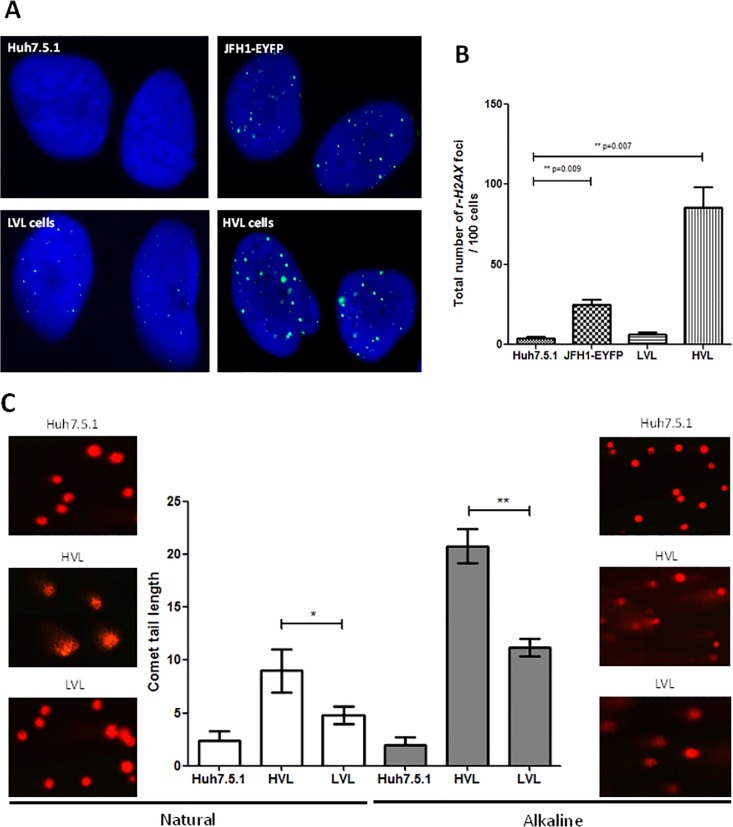
High levels of DNA damage in the HVL cells. (A) γ-H2AX foci formation was detected by immunocytochemical staining for γ-H2AX (green) and DNA counterstaining with DAPI (blue) in uninfected Huh7.5.1 cells, JFH1-EYFP-infected cells, LVL cells, and HVL cells. (B) The average number of γ-H2AX foci per 100 cells and the total count of 500 cells in three independent experiments. **P < 0.01. (C) The comet tail was detected by single cell gel electrophoresis at neutral pH for DSBs and alkaline pH for SSBs/DSBs in HVL, LVL, and uninfected-Huh7.5.1 cells. *P < 0.05, **P < 0.01.

To study the effect of the intracellular HCV viral load on the DNA damage response, first we performed a γ-H2AX foci formation assay and determined that significantly higher γ-H2AX foci numbers were present in the HVL cells compared to the LVL cells and unsorted JFH1-EYFP-infected cells (**Fig 2A and 2B**). These results directly demonstrated that HVL affected the DNA integrity. To determine the level of DNA strand breaks, single cell gel electrophoresis (SCGE, also known as the comet assay) was performed. Both the neutral assay for double-strand breaks (DSBs) and the alkaline assay for single/double-strand breaks (SSBs/DSBs) indicated that the HVL cells induced more DNA strand breaks than the LVL cells (**Fig 2C)**. These results demonstrated that the intracellular HCV viral load was highly associated with the amount of cellular DNA damage.

### Q-PCR array for damage-related gene expression in HVL and LVL cells

**Fig 3:**
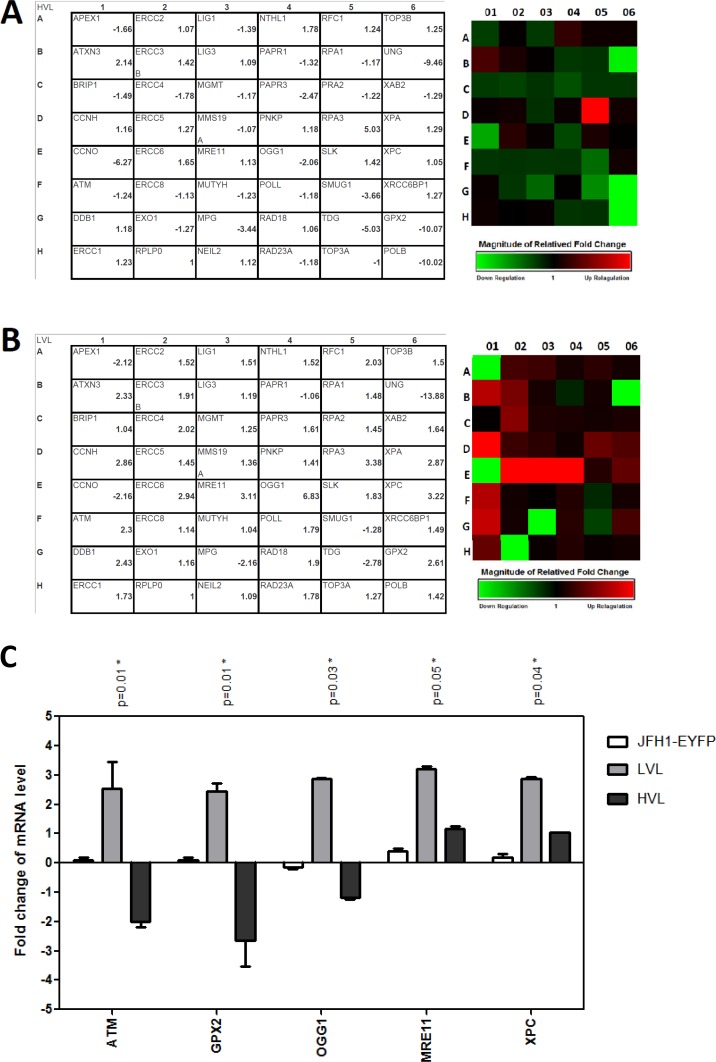
Different repair genes were identified in the HVL and LVL sorted cells. The 48 repair genes were analyzed, using a DNA Repair RT^2^ Profiler PCR Array kit, in (A) HVL cells and (B) LVL cells. The data were normalized to RPLP0 housekeeping gene levels and presented as the fold change. (C) The q-PCR of fold change expression of the *ATM*, *ERCC6*, *GPX2*, *MRE11*, *OGG1* and *XPC* mRNA levels in the HVL and LVL cells. Uninfected Huh7.5.1 cells served as the control group. *P < 0.05.

To identify damage-related gene alterations in the LVL and HVL cells, a q-PCR array was used to evaluate the expression of the essential genes of the three major DNA damage-repair mechanisms. A fold change greater than a two-fold threshold compared to the uninfected-Huh7.5.1 cells was used to define significant changes in the mRNA levels. The results showed that the *ATXN3*, *RPA3*, and *XPA* genes were up-regulated and the *APEX1*, *CCNO*, *MPG*, *SMUG1*, *TDG*, and *UNG* genes were down-regulated in both the HVL and LVL cells (**Fig 3A and 3B**). A significant down-regulation of the *PARP3* and *POLB* genes was observed in the HVL cells but not in the LVL cells. A significant up-regulation of the *ATM*, *CCNH*, *DDB1*, *ERCC6*, *GPX2*, *MRE11*, *OGG1*, *XPA*, and *XPC* genes was noted only in the LVL cells. These findings were confirmed by q-PCR for the individual genes **(Fig 3C)** using uninfected Huh7.5.1 cells as background. Notably, from the q-PCR array results, we confirmed that the *ATM*, *GPX2*, and *OGG1* genes were up-regulated in the LVL cells but inversely down-regulated in the HVL cells. These genes were not significantly different in the unsorted infected JFH1-EYFP cells compared to the uninfected Huh7.5.1 cells **(Fig 3C)**.

### Differential expression of DNA repair-associated proteins in the HVL and LVL cells

**Fig 4:**
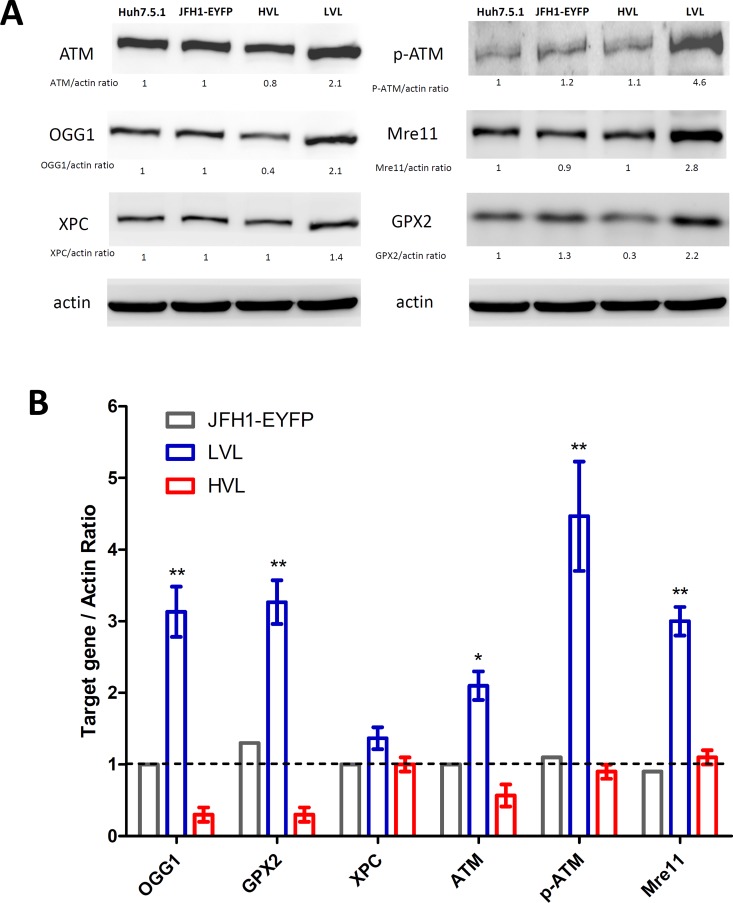
The protein expression levels of HVL and LVL sorted cells by western blotting. (A) The ATM, phospho-ATM, GPX2, MRE11, OGG1, and XPC levels were measured by western blot analysis. The figure is representative of three independent experiments. (B) The relative fold activity was quantified using AlphaImage software and normalized to the expression of the internal actin control. *P < 0.05, **P < 0.01.

To determine the protein expression levels and to assess significant differences in the q-PCR array data, western blot assays were conducted. These assays confirmed the opposing regulation of GPX2 and OGG1 in the LVL and HVL cells (**Fig 4A**). Used uninfected Huh7.5.1 cells were used as the control group, the data demonstrated similar q-PCR results and protein expression levels with the unsorted JFH1-EYFP infected cells. The protein data showed that the expression levels of GPX2 and OGG1 were significantly down-regulated in the HVL cells and inversely up-regulated in the LVL cells. Indeed, phospho-ser1981 ATM, total ATM, and MRE11 expression levels were significantly increased by more than two-fold in the LVL cells but were only slightly decreased or constantly expressed in the HVL cells, with the exception of XPC (**Fig 4B**). Nevertheless, the mRNA and protein results indicate that the intracellular HCV viral loads are directly correlated with DNA damage but present a paradoxical effect on distinct DNA damage repair activities.

### Repair ability of LVL cells following ionizing radiation

**Fig 5:**
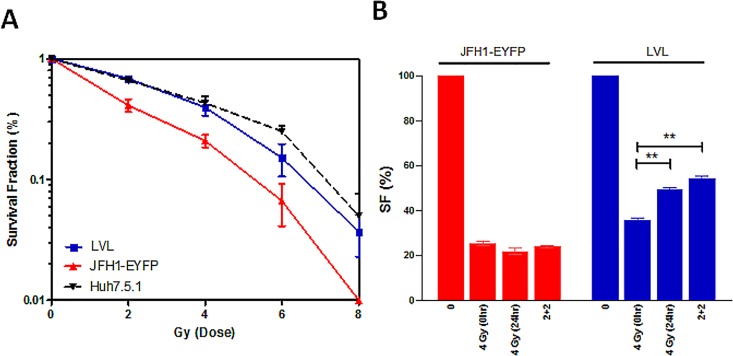
LVL cells increase resistance to RT. The survival curves following (A) single dose or (B) fractional irradiation were assayed by the colonogenic assay. **P < 0.01.

Cells with increased repair activity are more resistant to DNA damage inducible factors [14]. Hence, we evaluated the DNA damage repair efficiency of LVL cells following γ-ray irradiation. As expected, the HVL cells had a low colony formation efficiency due to a low plating efficiency and a low rate of cell proliferation. The survival curve for the colony formation assay after a single dose of irradiation showed that the LVL cells had a significantly higher survival rate than the unsorted JFH1-EYFP-infected cells (**Fig 5A**). Then, we examined the repair ability of the cells in response to sub-lethal radiation-induced DNA damage (SLDR; the radiation dose was split into two fractions and applied at a 24 h interval) and potentially lethal damage (PLDR; the cells were kept on the plates for 24 h after irradiation and then counted to assess colony formation) [13]. The results showed that the LVL cells but not the unsorted JFH1-EYFP-infected cells exhibited the ability to repair SLDR and PLDR (**Fig 5B**). These data reflect and extend the findings that damage-related genes are active in the LVL cells.

## Discussion

Several previous studies using HCV replicon cell lines demonstrated that HCV-induced oxidative damage and HCV-specific polyproteins induced genomic and chromosomal instability by impairing DNA repair [15–18]. However, HCV infection is not even distributed in the hepatocytes of chronic hepatitis C patients liver tissues [5, 19, 20]. Moreover, *in vitro* viral infection models cannot effectively isolate infected cells due to the lack of specific cell surface markers to clearly identify them. The process of intracellular immunofluorescence labeling of the HCV polyprotein is generally thought to degrade the RNA in the cells. In this study, we used an EYFP-tagged JFH1 virus and successfully and efficiently isolated infected cells with different viral loads by FACS. To the best of our knowledge, this is the first study to use FACS to delineate intracellular HVL and LVL populations and to investigate the effect of the intracellular viral load on host DNA damage responses. Using this approach, we clearly demonstrated that DNA breaks were highly present in the HVL cells and provided a direction for further studies on the effect of intracellular viral load on cell repair activities. The Huh 7.5.1 is so far the most stable, widely used cell line which can be infected by HCVcc. It’s not easy to infect the other cell lines with the HCVcc. Nevertheless, our results might not be translated to the other cell lines due to potential difference in the susceptibility to virus infection or the genetic heterogeneity. Although the cause of the differential viral presence in chronic hepatitis C patients’ hepatocytes remains unclear, we considered that genetic heterogeneity might not be the major issue affecting the susceptibility of each individual cell to virus infection in this study.

Previous studies showed that γ-H2AX was highly expressed in HCV NS2-expressing cells [15]. γ-H2AX plays an important role in regulating DNA damage repair pathways, and its phosphorylation is one of the signatures of DNA damage. In our model, the experimental results showed that the HVL cells expressed a high number of γ-H2AX foci and higher DNA damage levels for both DSBs and SSBs. γ-H2AX is attracted to replication-associated DSBs in S phase when host genomic replication is perturbed [21]. However, we did not observe changes in the cell cycle-associated protein CDK4, although we found a slight G1 arrest in the HVL cells. These results showing the association between the intracellular viral load and DNA breaks supported the findings of Higgs et al., who suggested that the expression of the entire complement of HCV proteins not only caused dsDNA breaks but also increased the ssDNA damage levels in a transgenic mouse model [16].

In chronic hepatitis, HCV produces more reactive oxygen species (ROS) and nitric oxide (NO) than other hepatitis viruses [18]. Patients with chronic hepatitis C have a greater than 80% chance of developing chronic diseases compared to hepatitis A, B and E patients [22]. An increase in the ROS levels by two to five orders of magnitude in liver tissues from CHC patients has also been reported [23]. We found that the viral loads were directly related to the cellular DNA damage levels but not to damage-related gene expression. The data from the LVL cells show that neither γ-H2AX foci nor DSBs are detectable, although SSBs are detectable in the alkaline comet assay. These results may be attributed to the effect of HVL-induced extracellular ROS, which activate the DNA repair pathway in adjacent LVL cells as a cell protection mechanism during viral infection.

The increased DNA break frequency or suppression of DNA repair pathways caused by viral proteins may increase the frequency of mutation and chromosomal rearrangements in HCV-infected cells [14]. The accumulated genetic alterations could lead to the formation of HCC. OGG1, ATM, and GPX2 are the key regulators that modulate the response to oxidative DNA damage in cells [24]. However, our results showed that HVL increased the frequency of DNA breaks and impaired DNA repair via the down-regulation of GPX2 and OGG1 expression at both the mRNA and protein levels. This result implies that highly infected cells may consider abundant viral replication as a DNA damage stress and restrict DNA damage repair signal transduction, which may ultimately induce apoptosis as part of the host defense mechanism against virus infection. In contrast to the suppression observed in the HVL cells, ATM, GPX2, MRE11, and OGG1 gene expression levels were markedly increased in the LVL population, which is responsible for the repair of oxidative stress-induced lesions and is associated with the resistance to irradiation damage by increased SLDR and PLDR in the LVL cells. This finding could account for the decrease in detectable strand breaks in the LVL population relative to the HVL population. Oxidative stress in the HVL population produces lesions that are beyond repair. The cells favor a cell death program over a survival/repair situation, which may explain why these cells do not up-regulate their repair-associated genes to the extent observed in the LVL cell population.

In conclusion, the present study established a FACS protocol to study the effects of different intracellular viral loads in HCV-infected cell populations. The results demonstrated that the different viral loads in the cells presented different levels of DNA damage and capacities for damage-related gene expression. Our findings highlight the important role of viral loads in the study of gene expression profiles in viral infection-associated research.

## Supporting Information

S1 Fig Delineating high and low viral loads cell populations for JFH1-EYFP infected cells on day 5 post-infection by the flow sorter system. (A) FACS analysis of a single cell suspension for Forward Scatter (FSC) and Side Scatter (SSC). (B) Two parameter histogram Dot Plots displaying FL1-EYFP on the x axis and SSC on the y axis for LVL gated on the lowest 20% EYFP-intensity population and HVL gated on the highest 20% EYFP-intensity population. (C) The EYFP Dot Plot shows the LVL FL1-EYFP intensity after the flow sort. (D) The EYFP Dot Plot shows the HVL FL1-EYFP intensity after the flow sort.
(PDF)

S2 Fig Cell viability of the total JFH1-EYFP-infected cells and the sorted low and high viral load cells was assessed by staining with PI and analyzed by flow cytometry analysis. The x-axis indicates the intensity of viral EYFP fluorescence signaling. The y-axis indicates the PI intensity. All cell populations presented less than 10% PI-positive cells.
(PDF)

S3 Fig Cell cycle analysis of the total JFH1-EYFP-infected cells and sorted low and high viral load cells assessed by the PI labeling DNA assay and analyzed by flow cytometry. The percentages of the indicated G1, S, and G2 phase contents in all of the cell populations is shown.
(PDF)

S1 Table Primer sequences for Q-PCR Array and Q-PCR target genes. All gene names and accession number were obtained from GenBank (National Center for Biotechnology information 2009).
(PDF)
