# Supplementary figures and images for: The Paradoxical Effects of Different Hepatitis C Viral Loads on Host DNA Damage and Repair Abilities
Source: PLoS One. 2017 Jan 4;12(1):e0164281. doi: 10.1371/journal.pone.0164281 (PMC5215444; doi:10.1371/journal.pone.0164281)

S1 Fig

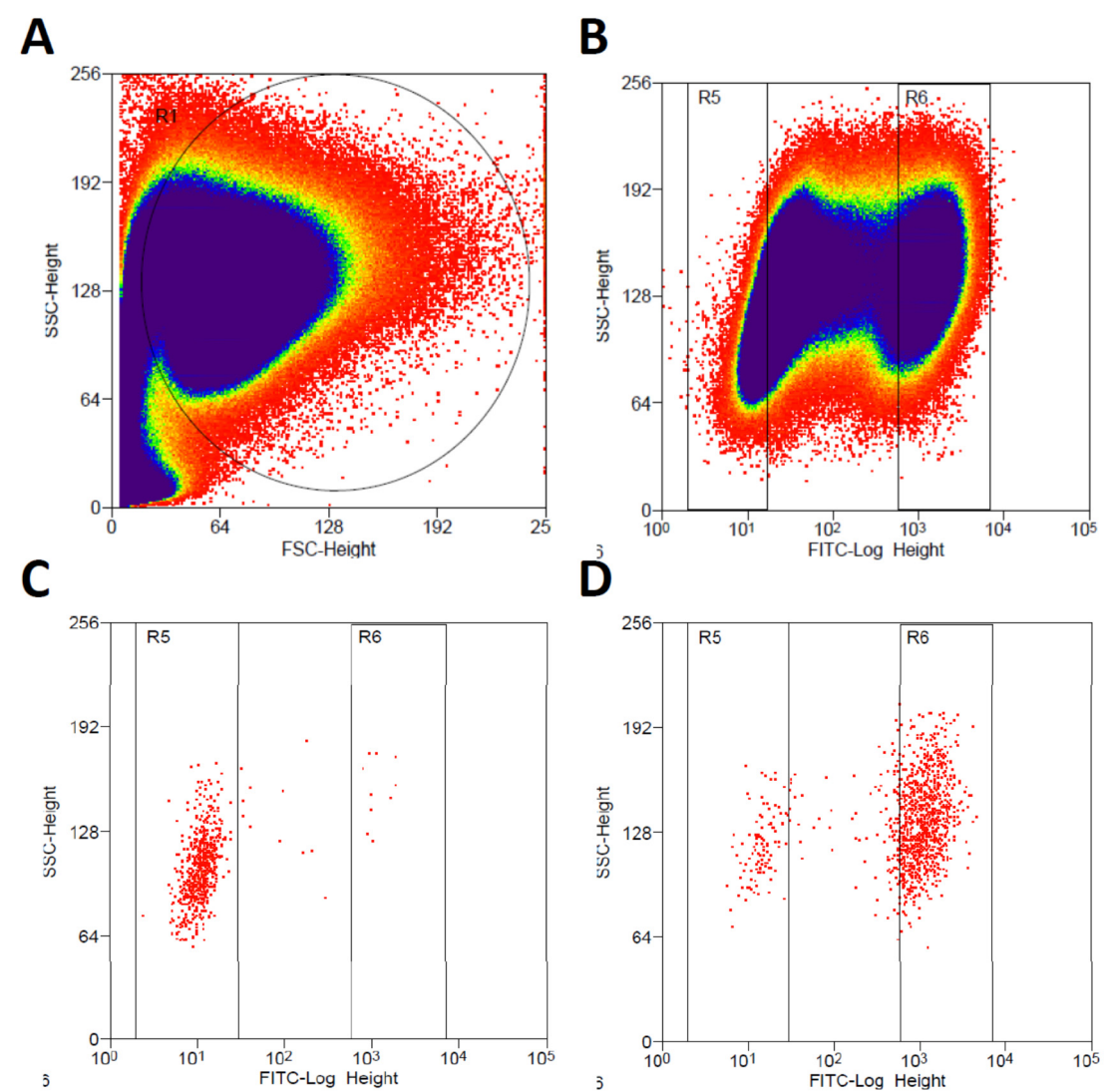

Supplement: S1 Fig — (A) FACS analysis of a single cell suspension for Forward Scatter (FSC) and Side Scatter (SSC). (B) Two parameter histogram Dot Plots displaying FL1-EYFP on the x axis and SSC on the y axis for LVL gated on the lowest 20% EYFP-intensity population and HVL gated on the highest 20% EYFP-intensity population. (C) The EYFP Dot Plot shows the LVL FL1-EYFP intensity after the flow sort. (D) The EYFP Dot Plot shows the HVL FL1-EYFP intensity after the flow sort. (PDF) [file pone.0164281.s001.pdf]

S2 Fig

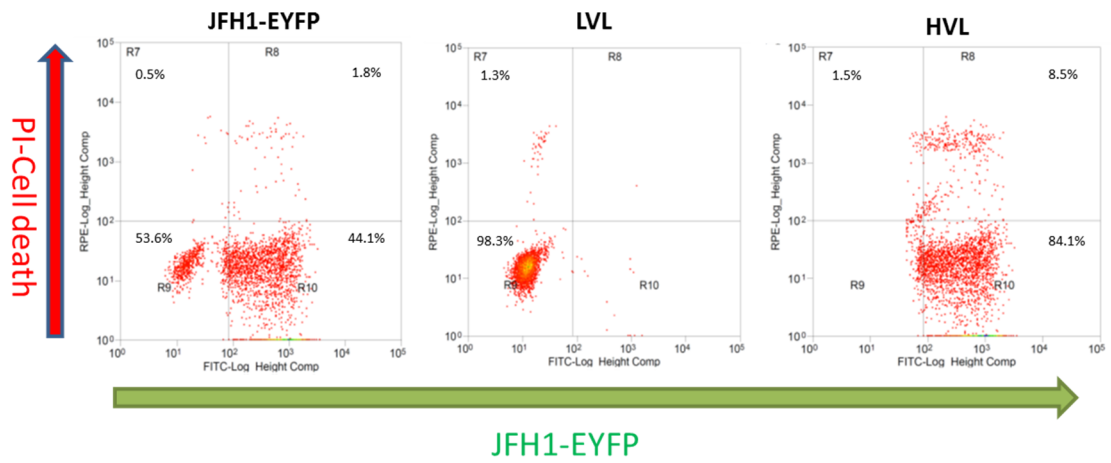

Supplement: S2 Fig — The x-axis indicates the intensity of viral EYFP fluorescence signaling. The y-axis indicates the PI intensity. All cell populations presented less than 10% PI-positive cells. (PDF) [file pone.0164281.s002.pdf]

S3 Fig

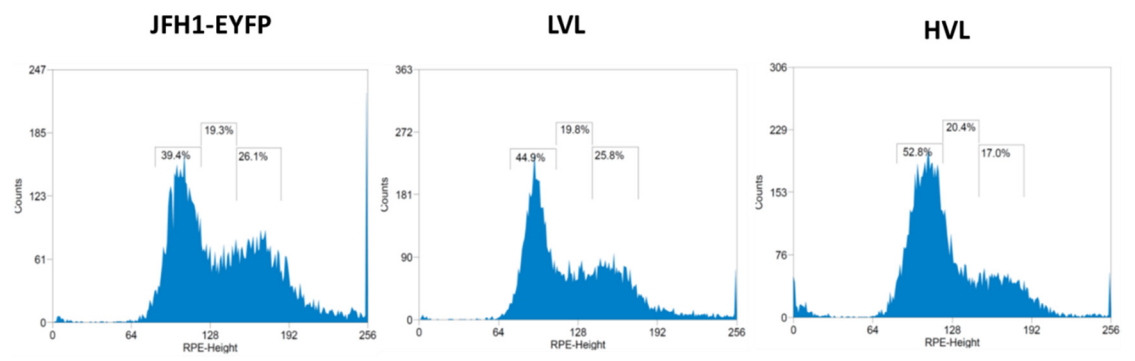

Supplement: S3 Fig — The percentages of the indicated G1, S, and G2 phase contents in all of the cell populations is shown. (PDF) [file pone.0164281.s003.pdf]
