# Supplementary material for: The Paradoxical Effects of Different Hepatitis C Viral Loads on Host DNA Damage and Repair Abilities
Source: PLoS One. 2017 Jan 4;12(1):e0164281. doi: 10.1371/journal.pone.0164281 (PMC5215444; doi:10.1371/journal.pone.0164281)
Supplement: S1 Table — All gene names and accession number were obtained from GenBank (National Center for Biotechnology information 2009). (PDF) [file pone.0164281.s004.pdf]

S1 Table.

GenBank	Symbol	Description	Primer
NM_080649	APEX1	Apurinic/apyrimidinic endodeoxyribonuclease 1	AGGCCGAGGTCTGGTACGA
			GGAACTTGCGAAAGGCTTCA
NM_004993	ATXN3	Ataxin 3	TGGTGCGTTCCTAAACTCTG
			GGGTTGCAACAAAGCTGTAA
NM_000051	ATM	Ataxia telangiectasia mutated	TCCATCGTCCACTGGTCTTTC
			GTCGGCAGCTAAAGGACTCATG
NM_032043	BRIP1	BRCA1 interacting protein C-terminal helicase 1	TCGGGAATCAGCAAGTTACA
			ATGAGGCTACAGCACACAGC
NM_001239	CCNII	Cyclin II	ATTGCATTGACGGATGCTTA
			AGCCCTGGAGGCACTAGATA
NM_021147	CCNO	Cyclin O	TCCCGTAGCCTCAGAAGAGT
			TTTACAACCTGCAGCTGACC
NM_001923	DDB1	Damage-specific DNA binding protein 1	TCTCACATGATTCCAGCCAT
			AGCTTCCTTTCAGCCAAAGA
NM_001983	ERCC1	Excision repair cross-complementing rodent repair deficiency, complementation group 1 (includes overlapping antisense sequence	AGTCAGACCCTCCTGACCAC
			CCAGATCTTCTCTTGATGCG
NM_000400	ERCC2	EExcision repair cross-complementing rodent repair deficiency, complementation group 2	CCCATACTTCCTTGCTCGAT
			AGTTCCTTGGACACCAGGTC
NM_000122	ERCC3	Excision repair cross-complementing rodent repair deficiency, complementation group 3 (xeroderma pigmentosum group B complementing)	TTCTGTGGAGCAGTGGAAAG
			CCAGCATGGAGTAGGTGCTA
NM_005236	ERCC4	Excision repair cross-complementing rodent repair deficiency, complementation group 4	TGACATTGAACCCGTGACTT
			GGAGATGCACTGGCTGTAGA
NM_000123	ERCC5	Excision repair cross-complementing rodent repair deficiency, complementation group 5	GGAAGATTCGCTCCATGAAT
			AGTAGCAGCGATCCGTTCTT
NM_000124	ERCC6	Excision repair cross-complementing rodent repair deficiency, complementation group 6	TCCCAGAGCACTGAAACAAG
			ACATCATGGTCTGCTCCAAA
NM_000082	ERCC8	Excision repair cross-complementing rodent repair deficiency, complementation group 8	TCCTGGTCTCCACGTTATGA
			GATGCTCTTCTCACATCCCA
NM_130398	EXO1	Exonuclease 1	AGGAATGTGCAGACAGCTTG
			GACTTTGCATGCCTTTGCTA
NM_002083	GPX2	Glutathione peroxidase 2	GCCTCAAGTATGTCCGACCTG
			GGAGAACGGGTCATCATAAGGG
NM_000234	LIG1	Ligase I, DNA, ATP-dependent	TCTGGTTACAATCCTGCCAA
			AGCAGACACCTCCTCGATCT
NM_002311	LIG3	Ligase III, DNA, ATP-dependent	ACCCACCAGTTCTCCACTGT
			AGCACCCAGCTCTGAAAGAT
NM_002412	MGMT	O-6-methylguanine-DNA methyltransferase	GATGGATGTTTGAGCGACAC
			AGGACACTGCCACTTCCTTT
NM_022362	MMS19	MMS19 nucleotide excision repair homolog	GCTGAGGACCTCCTTGACTC
			AGTTTCATGTCCGGTTCACA
NM_005590	MRE11A	MRE11 homolog A, double strand break repair nuclease	TGATGAAGTCCGTGAGGCTATG
			AGAAGCAGACTCCTCTGACTGAGAT
NM_012222	MUTYH	MutY homolog	CGCCCAGGAGTTAGACTAGG
			GAGCTCTCCATCCTCTCTGG
NM_002434	MPG	N-methylpurine-DNA glycosylase	CCGCAGCATCTATTTCTCAA
			TCGGAGTTCTGTGCCATTAG
NM_145043	NEIL2	Nei endonuclease VIII-like 2	ATTGAGGCTGAGGAGCTTGT
			CTGACTGCCTGCACTTGAAT
NM_002528	NTHL1	Nth endonuclease III-like 1	AGCAACAGCTGGTCAACATC
			CCTGGTACCTGCGTACCTTT
NM_001618	PARP1	Poly (ADP-ribose) polymerase 1	TCTGGACTGGAACACTCTGC
			TCCTTTAACGATGTCCACCA
NM_005485	PARP3	Poly (ADP-ribose) polymerase family, member 3	TTCTCCCAGAGCGAGTACCT
			GTACCAGAGATGGGCAGGAT
NM_007254	PNKP	Polynucleotide kinase 3'-phosphatase	GGGAAGTCCACCTTTCTCAA
			GTCTCACACGTGGTCACACA
NM_002542	OGG1	8-oxoguanine DNA glycosylase	GAGGCCGTGCGCAAGTA
			CCCCAGTGGTGATACAGTTGAG
NM_013274	POLL	Polymerase (DNA directed), lambda	GCCAAAGCCTACAGTGTTCA
			AAGCTCTTGAGGGCATTGAT
NM_020165	RAD18	RAD18, E3 ubiquitin protein ligase	AAGCAAATTCAGCCCTCAAA
			TTAGCCTCTGAGGGATCTGG
NM_005053	RAD23A	RAD23 homolog A	TGACGGGCTCTGAGTATGAG
			TGTTGTAGCTGGCTCTCAGG
NM_002913	RFC1	Replication factor C (activator 1) 1	AAGGCTAGGAATTTGGCTGA
			TAGGAGTTTGTTGGCACAGC
NM_002945	RPA1	Replication protein A1	TGGCCACAGTGGTGTATCTT
			CACAGCGGTACAATCCATTC
NM_002946	RPA2	Replication protein A2	GAGCACCTATCAGCAATCCA
			TTGCTGGCATGAAGCTATTC
NM_002947	RPA3	Replication protein A3	CATGGTGGACATGATGGACT
			ACAGGCTTGTCGATGAATTG
NM_001002	RPLP0	Ribosomal protein, large, P0	ACTCTGCATTCTCGCTTCCT
			CTCGTTTGTACCCGTTGATG
NM_002690	POLB	Polymerase (DNA directed), beta	TGTGGCAGTTTCAGAAGAGG
			TCTGAAGTGAAGCTGGGATG
NM_014720	SLK	STE20-like kinase	TCCGAGAATTGATTGCAGAG
			CTTGCAGGTATTGGCAGAGA
NM_014311	SMUG1	Single-strand-selective monofunctional uracil-DNA glycosylase 1	GTGTGGGAAAGAAGCTAGGC
			ATTTGGTTGAGCCCAATCTC
NM_003211	TDG	Thymine-DNA glycosylase	GGCTGGGTACAGACACACAC
			ATCCCAGACTCCAAGGACAC
NM_004618	TOP3A	Topoisomerase (DNA) III alpha	CTGGAGTTTGTTTGCTGCAT
			GTCCATCCTGTTCAGGGACT
NM_003935	TOP3B	Topoisomerase (DNA) III beta	AGCATCCCTGTGCATATCAA
			CACCAGCTCTGCATCAATCT
NM_003362	UNG	Uracil-DNA glycosylase	CCGCAACCGAATTAACACTA
			TGAGTTCCAAACGAAAGTCG
NM_020196	XAB2	XPA binding protein 2	CTCCGACACGAGAACTACGA
			CATGGACCAGACCTTCAGTG
NM_000380	XPA	Xeroderma pigmentosum, complementation group A	AAAGGAAGTCCGACAGGAAA
			TGATGAACAATCGTCTCCCT
NM_004628	XPC	Xeroderma pigmentosum, complementation group C	AGAGCCCATTTATGGACAGG
			GGCATACAGAGGGTGGTTCT
NM_033276	XRCC6BP1	XRCC6 binding protein 1	GAGTGGTCACACACGAGCTT
			ACGCCAAATGTCTGATGTTG
